# Supplementary material for: The limited reach of fake news on Twitter during 2019 European elections
Source: PLoS One. 2020 Jun 18;15(6):e0234689. doi: 10.1371/journal.pone.0234689 (PMC7302448; doi:10.1371/journal.pone.0234689)
Supplement: S1 File — (PDF) [file pone.0234689.s001.pdf]

The Limited Reach of Fake News on Twitter during 2019 European Elections

Matteo Cinelli[1], Stefano Cresci[2], Alessandro Galeazzi[3,*], Walter Quattrociocchi[4], Maurizio Tesconi[2]

1 ISC CNR, Rome, Italy
2 IIT CNR, Pisa, Italy
3 University of Brescia, Italy
4 Ca'Foscari Univerisity of Venice, Italy

* a.galeazzi002@unibs.it

Supporting Information

1 Language Analysis

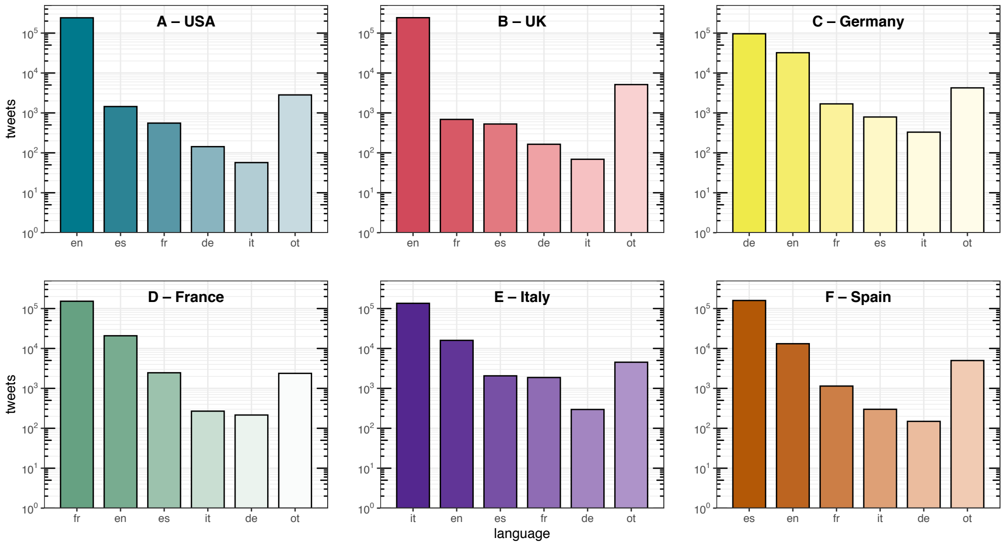

Fig 1. Per country distribution of languages in the tweets of our dataset.

In order to give more details about the impact of language barriers on the debate among nations, we provide more details about the languages used in the six more relevant nations in our analysis. Figure 1 shows that the national language is indeed the most frequent in the tweets of the respective country; nonetheless other languages, and especially English in non-English speaking countries, is well represented within the Twittersphere. Table 1 provides more details concerning the usage of national and foreign languages in different countries.

In summary, we can conclude that despite the national language is the most used in each countries also other languages are quite well represented and therefore the impact of language barriers is present but it is not the unique element that determines the lack of inter-connections among countries.

country	total	home lang	other langs	home lang from outside	home total ratio	other home ratio	outside total ratio
UK	249,923	243,342	6,581	323,857	0.9737	0.0270	1.2958
USA	246,760	241,735	5,025	325,464	0.9796	0.0208	1.3190
Spain	178,815	159,091	19,724	7,286	0.8897	0.1240	0.0407
France	178,593	152,482	26,111	5,943	0.8538	0.1712	0.0333
Italy	159,814	135,128	24,686	1,024	0.8455	0.1827	0.0064
Germany	135,481	96,187	39,294	967	0.7100	0.4085	0.0071

Table 1. The column **country** refers to the geolocation of the tweets at national level. The column **total** is the total number of tweets located in the respective country. The column **home lang** reports the number of tweets using the national language of the country. The column **home lang from outside** reports the number of tweets made in different countries that the national language of the country belonging to the respective row. The other columns report the ratio deriving from the previous columns.
