# Supplementary material for: The limited reach of fake news on Twitter during 2019 European elections
Source: PLoS One. 2020 Jun 18;15(6):e0234689. doi: 10.1371/journal.pone.0234689 (PMC7302448; doi:10.1371/journal.pone.0234689)
Supplement: Attachment — Submitted filename: Response_to_Reviewers.pdf [file pone.0234689.s002.pdf]

Response to Reviewers

April 11, 2020

Introduction

We first want to thank the two reviewers for their attention and their comments which, we believe, allowed us to improve the exposition of the results. In what follows, we address each reviewer's comments separately, reviewer 1 in section 1 and reviewer 2 in section 2.

1 Reviewer 1

1.1 Q1

1. I am not sure whether the 2019 European Parliament election is a proper case for the authors' purpose. Are there instances of known fake news or propaganda that was spread online and became a serious issue during the 2019 European Parliament election like those that emerged during Brexit and the US election in 2016? I am concerned that this election might represent an exceptional case.

ANSWER

We understand the concern of the reviewer and in the following lines we will motivate our case study. During the electoral campaign, concerns about the impact of fake news on the upcoming European election were risen by several news outlets[1] and misinformation have been monitored, also thanks to the effort of NGOs, in different platforms[2]. The EU itself started a joint and coordinated action on misinformation mitigation[3]. Based on what happened during Brexit and the US 2016 election, also EU leaders encouraged the adoption of measures at the European level to counteract the diffusion and impact of Fake News[4]. Starting from these premises, our study aims to assess the reach of fake news during European Elections.

We agree with the reviewer that not all public events attract fake news in the same way. Nonetheless, we think it is relevant and useful to measure their spread in different scenarios. In our present study, we found that fake news did not spread in the context of the 2019 European Parliament election as much as they did during the Brexit debate or the 2016 US elections, but we think that our case of study is relevant to the question and that our results can be useful to assess the effectiveness of the countermeasures to fake news diffusion deployed by Twitter. As additional evidence of the importance of our research

[1] https://www.bbc.com/news/blogs-trending-48296557
[2] https://secure.avaaz.org/campaign/en/disinfo_network_report/
[3] https://ec.europa.eu/digital-single-market/en/tackling-online-disinformation
[4] https://www.reuters.com/article/us-eu-disinformation/eu-leaders-to-warn-of-cyber-fake-news-threat-to-may-elections-idUSKCN1R11QM

question, other recent papers (e.g. Pierri, Artoni, and Ceri (2020)) investigated fake news – although with a different approach and with reduced breadth – during the 2019 European Parliament elections.

In the introduction of the paper, to provide evidence of the concern related to EU elections, we added the following sentences:

During the electoral campaign, concerns about the impact of fake news on the upcoming European election were risen by several news outlets[5] and misinformation have been monitored, also thanks to the effort of NGOs, in different platforms[6]. The EU itself started a joint and coordinated action on misinformation mitigation[7]. Based on what happened during Brexit and the US 2016 election, also EU leaders encouraged the adoption of measures at the European level to counteract the diffusion and impact of Fake News[8]. Additional evidence of the potential impact of misinformation during European Election motivated studies at national level such as Pierri et al. (2020). Starting from these premises, our study aims to assess the reach of fake news during European Elections.

1.2 Q2

The second comment is related to the previous one. The current manuscript overstates the peripheral role of fake news accounts and the effectiveness of the ban on suspicious accounts. First, 49 fake news accounts in this study were depicted from the lists of authoritative reports, which is a small subset of potential malicious users. Second, the authors did not examine the effect of the ban using any quantitative methodology. They have to be careful about concluding that they have. In actuality, a large volume of malicious accounts and fake news have been reported in the case of the 2019 Hong Kong protests.

ANSWER

The identification of fake news and misinformation online is an open problem and different approaches to it can lead to different results, as pointed out in Ruths (2019). The labelling of fake news and fake news accounts is beyond the scope of this paper and we think it would not be fair to rely on a list of malicious users we made ourselves. Rather, relying on external sources (i.e. fact-checking agencies) is the best option available to reduce possible biases.

The second paragraph of section Materials and methods reports the list of sources used for fake news accounts identification.

Finally, the last sentence of the Introduction and of the Conclusions have been rephrased in a more factual way underlining that, taking into account both the initial concerns and our results, the countermeasures implemented by Twitter *may have reduced* the spreading of misinformation.

[5] https://www.bbc.com/news/blogs-trending-48296557
[6] https://secure.avaaz.org/campaign/en/disinfo_network_report/
[7] https://ec.europa.eu/digital-single-market/en/tackling-online-disinformation
[8] https://www.reuters.com/article/us-eu-disinformation/eu-leaders-to-warn-of-cyber-fake-news-threat-to-may-elections-idUSKCN1R11QM

- However, the lack of interactions between fake news accounts and others demonstrated that their user base is confined to a peripheral portion of the network, suggesting that the countermeasures taken by Twitter, such as suspension or ban of suspicious accounts, were likely effective in keeping the Twittersphere clean.
- However, the lack of interactions between fake news accounts and others demonstrated that their user base is confined to a peripheral portion of the network, suggesting that the countermeasures taken by Twitter, such as suspension or ban of suspicious accounts, might have been effective in keeping the Twittersphere clean.
- Finally, the limited and bounded contribution that disinformation outlets had on the overall interactions may suggest that the strategies employed by Twitter to counteract the spreading of disinformation – that is, the ban or suspension of suspicious accounts – are indeed effective and can help to preserve the integrity of the Twittersphere.
- Finally, the limited and bounded contribution that disinformation outlets had on the overall interactions suggests that the strategies employed by Twitter to counteract the spreading of disinformation – that is, the ban or suspension of suspicious accounts – may have had a mitigation effect on the spreading of fake news thus preserving the integrity of the Twittersphere.

1.3 Q3

The authors analyzed only the number and patterns of interactions (i.e., mentions, replies, retweets, URLs) among 80 account types and did not analyze tweet texts. These are, therefore, unknown content and sentiment-level differences in fake news accounts as well as other account types. FYI, the following paper is relevant to this study:

Stella, M., Ferrara, E. De Domenico, M. Bots increase exposure to negative and inflammatory content in online social systems. PNAS (2018).

ANSWER

In the paper, we indeed focused on the volume of interactions among different class of users and not on their content. This was a research design choice, since the contributions of the different actors on the public debate can be quantified independently of the contents. Hence, we consider the sentiment analysis beyond the scope of this paper and it would probably require a lot of fine tuning in order to be explanatory. Indeed, such kind of analysis can also be inaccurate due to the presence of satire, ironic contents and trolls.

Finally, we thank the reviewer for the reference suggestion. The analogy between the behaviour of fake news and automated accounts is an interesting point that we discuss in the Result and Discussion as follow:

Interestingly, the behavior of fake news accounts is akin to that of automated accounts as shown by the authors of Stella, Ferrara, and De Domenico (2018) during the Catalan Referendum: both fake news and automated accounts tend to target popular users in order to increase their relevance and impact in the public debate.

2 Reviewer 2

2.1 Q1

The authors identified five classes (showbiz, social media, sports, trademark vip) in accounts based on most interacted with fake, official, and politicians accounts. How did the authors define “most” interacted? Furthermore, since the result sounds similar to Stella(2018), the authors should discuss the difference. M. Stella, E. Ferrara, and M. De Domenico, “Bots increase exposure to negative and inflammatory content in online social systems,” Proc. Natl. Acad. Sci., vol. 115 (49) pp. 12435–12440 (2018).

ANSWER

Given our initial dataset of Official, Politicians and Fake news accounts, we enlarged such dataset by including the accounts with more than 1M followers that interacted with them. Therefore, our definition of most popular is having more than 1M followers. In the paper to provide a quantitative measure of ”most popular” we rephrased such sentences in the following way:

In section Results and Discussion the following sentence:

- *This is due to the fact that we integrated our initial set of accounts with the most popular accounts that interacted with them.*

  has been turned into:

- This is due to the fact that we integrated our initial set of accounts with a subset of popular accounts (more than 1M followers) that interacted with them.

In subsection Data collection we changed the following sentence:

- *After this step, we also manually classified a small subset of the most popular accounts that interacted with those of our initial lists in the considered time period.*

  has been turned into:

- After this step, we also manually classified a small subset of popular users (more than 1M followers) that interacted with those of our initial list in the considered time period.

Finally, we thank the reviewer for the reference suggestion. The analogy between the behaviour of fake news and automated accounts is an interesting point that we discuss in the Result and Discussion as follow:

Interestingly,the behavior of fake news accounts is akin to that of automated accounts as shown by the authors of Stella et al. (2018) during the Catalan Referendum: both fake news and automated accounts tend to target popular users in order to increase their relevance and impact in the public debate.

2.2 Q2

Fig.1 My main concern is comparing account nationalities and classes (official, sports, etc.). I am not sure there is a significant difference between them. For example, in a country-by-country context, Oceania accounts have 0.38% in total, perhaps only 3 accounts, which makes it difficult to discuss. In a class-by-class context, official and political have more than 300 accounts, while trademark and social media have fewer than 10. Furthermore, it's also not clear the criteria to separate five classes.

ANSWER

We understand the concern of the reviewer. The presence of differences in the frequency of certain classed is due to nature of the data collection performed. In fact, as reported in the paper (see Sections **Results and Discussion** and **Data Collection**), we initially selected a set of politicians, official news outlets and fake news outlets to study the information flow and the spread of fake news. Then, we added popular users ($> 1M$ followers) with a different public role that interacted with our initial set of accounts. We did this in order to also consider accounts that could have been exploited by fake news outlets for attracting the attention of the general audience. Clearly, these new users needed to be classified into new categories and their distribution and location varies from class to class and from place to place. We wanted to highlight the geographical and class distribution of the accounts to give an overview of the dataset and to pinpoint that the debate rarely cross national borders, probably for both limited interest and for language barriers. Notably, the limited presence of accounts belonging to some classes (e.g., social media, trademarks, VIPs, sport) is also easily explained by considering that such accounts have no particular interest in discussing about the EU Parliament elections, contrarily to disinformation and authoritative news outlet and to politicians. As such, they appear as underrepresented in our dataset.

Furthermore, we remark that the main findings of our study are related to the disinformation, authoritative, and the politicians classes of users, which are all well represented in our dataset. The remaining classes were added for completeness, although they do not represent the focus of our work.

2.3 Q3

line.95 Fig.2 - This may be a first signal that the interactions rarely cross national borders.

I am not sure it is because of its national borders. Did the authors consider language differences? If all accounts that considered here use English, the authors should mention that, and if not, it is possible that the limited interactions occurred because of languages, too.

ANSWER

We did not want to infer that there is little exchange among nations because of the national borders, but rather we just noted this. We are sure that language barriers exists and that they play a role in information exchange among nations, however quantifying this

role is beyond the scope of our study. Nonetheless, we appreciate the reviewer's suggestion and we included both Table T1 and Figure S1 in our Supporting Information to show the distribution of tweet languages, as reported by the Standard Twitter API, per country, in our dataset. For clarity, the same material are also added below.

Figure 1 shows that the national language is indeed the most frequent in the tweets of the respective country; nonetheless other languages, and especially english in non-english speaking countries, is well represented within the Twittersphere. Table 1 provides more details concerning the usage of national and foreign languages in different countries.

In summary, we can conclude that despite the national language is the most used in each countries also other languages are quite well represented and therefore the impact of language barriers is present but it is not the unique element that determines the lack of inter-connections among countries.

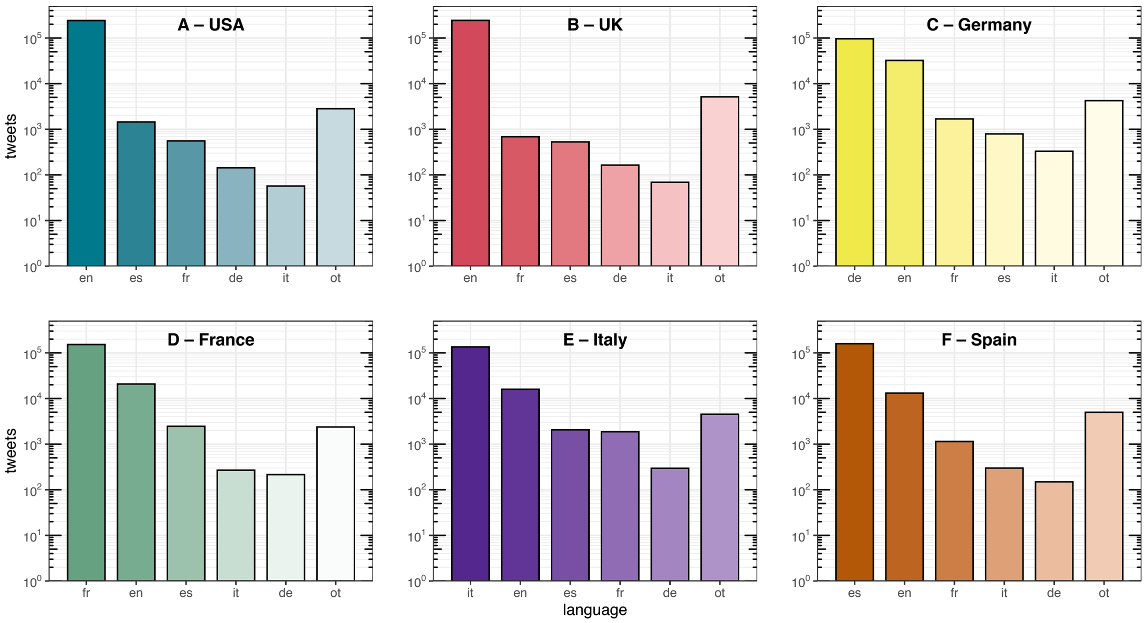

Figure 1: Per country distribution of languages in the tweets of our dataset.

country	total	home lang	other langs	home lang from outside	home total ratio	other home ratio	outside total ratio
UK	249,923	243,342	6,581	323,857	0.9737	0.0270	1.2958
USA	246,760	241,735	5,025	325,464	0.9796	0.0208	1.3190
Spain	178,815	159,091	19,724	7,286	0.8897	0.1240	0.0407
France	178,593	152,482	26,111	5,943	0.8538	0.1712	0.0333
Italy	159,814	135,128	24,686	1,024	0.8455	0.1827	0.0064
Germany	135,481	96,187	39,294	967	0.7100	0.4085	0.0071

Table 1: The column **country** refers to the geolocation of the tweets at national level. The column **total** is the total number of tweets located in the respective country. The column **home lang** reports the number of tweets using the national language of the country. The column **home lang from outside** reports the number of tweets made in different countries that the national language of the country belonging to the respective row. The other columns report the ratio deriving from the previous columns.

2.4 Q4

line.168 - the engagement, computed as the number of retweets, generated by the different classes of actors.

About the definition of the "engagement," if the authors computed as the number of retweets generated by the different classes of actors, how did they separate each retweet by the different classes of actors? If they simply counted a number of retweets, I suggest using the word "retweets" instead of "engagement" to avoid confusion. Furthermore, did they include mention and reply?

ANSWER

We apologize for the inconsistency pointed out by the reviewer. In the above-mentioned paragraph, we are referring to the engagement *obtained* by the different classes of actors, rather than the engagement they *create*. In particular, each actor produces tweets, and each tweet obtains a certain engagement that derives from the interactions (e.g., retweets) of other users with that tweet. We can thus aggregate the engagement obtained by all tweets of a given actor, to have an indication of the engagement obtained by that actor. Similarly, we can aggregate the engagement obtained by all tweets from actors of a given class (e.g., politicians, fake news outlets, etc.), to have an indication of the engagement obtained by that class of actors.

Regarding our wordings, we better clarified that our proxy for the engagement of a tweet is its number of retweets and we additionally added this information in all figures depicting engagement/retweet counts (both in the figures and in the captions).

Finally, Standard (i.e., free) Twitter APIs do not provide information about the number of replies to a given tweet[9] or about the number of mentions to a given user[10]. Because of this reason, we leveraged retweet counts as our proxy for the engagement obtained by tweets. Furthermore, since the number of retweets provide an indication for how much a message spread, it arguably represents a good indicator for investigating the *reach* of fake vs authoritative news, which is the goal of our study.

In the revised version of our manuscript we have rephrased all sections related to our analysis of the engagement obtained by the different (classes of) actors. In particular, we have made clear that we are analyzing the engagement *obtained* by those actors, and we have better clarified why our proxy for the engagement is the number of obtained retweets.

2.5 Q5

line.175 - popularity, computed as the number of followers, of the accounts belonging to the different classes of actors.

Similar to the "engagement," if the authors computed as the number of followers, I suggest using the word "followers" instead of "popularity" to avoid confusion.

ANSWER

[9] `https://developer.twitter.com/en/docs/tweets/data-dictionary/overview/tweet-object`

[10] `https://developer.twitter.com/en/docs/tweets/data-dictionary/overview/user-object`

We thank the reviewer for the observation. In order to avoid confusion we have clarified that our proxy for the popularity of an accounts is its number of followers. In addition, we also added this information in all figures depicting popularity/followers counts (both in the figures and in the captions).

2.6 Q6

line.172 Fig.5 - Figure 5 shows that the engagement of disinformation outlets is dominated by that of all other classes

I do not understand Figure 5 fully. What does "dominate" mean here? The vertical axis of Figure 5 is "users," but isn't it a "tweet"? Since the total number of social media accounts is 8, the vertical axis of Figure 5 for orange one seems strange.

ANSWER

There was a typo in the label of the y axis of Figure 5, which should report "tweets" instead of "users", as correctly explained in the figure caption. We fixed this issue in our revised manuscript.

Regarding the interpretation of the figure, we wanted to highlight that the overall engagement obtained by tweets of disinformation outlets is lower than that obtained by all other classes of actors. This is shown in Figure 5 by considering that the distribution of the engagement for all classes reaches larger values on the x axis, with respect to the distribution for disinformation outlets. Following the reviewer's suggestion, in our revised manuscript we have made this point clearer by expanding and rewording the explanation and discussion of the figure.

2.7 Q7

Data availability: I checked the pointed web site and found a list of 863 accounts, but I could not find information about the interactions such as reply, mention, and others. https://github.com/cinhelli/Limited-Reach-Fake-News-Twitter-2019-EU-Elections

ANSWER

In order to comply with Twitter's Terms of Service[11], we originally planned to share only the IDs of the accounts involved in our study, as done in the majority of research papers that are based on Twitter data. With the user IDs, interested researchers could reconstruct the dataset by querying Twitter APIs. However, after reading the reviewer's comment, we thought it could be more useful to share the full *pseudonymized* dataset. We have thus updated the GitHub page[12] where the dataset is hosted by uploading the full pseudonymized version. Pseudonymization is obtained by hashing user IDs and by removing all other information that could be used to retrieve the original data (e.g., tweet IDs and texts). The shared dataset now explicitly features all interactions between our users, as well as information about the number of followers of such users, the number of

[11] `https://developer.twitter.com/en/developer-terms/agreement-and-policy`
[12] `https://github.com/cinhelli/Limited-Reach-Fake-News-Twitter-2019-EU-Elections`

retweets and the language of tweets, and geographic information. We thank the reviewer for this suggestion. (SCRIVIAMOLO ANCHE NEL PAPER MAGARI)

In subsection Data Collection of the paper we reported the follwing sentence: A pseudonymized version of our dataset is publicly available on GitHub[13].

References

Pierri, F., Artoni, A., & Ceri, S. (2020). Investigating italian disinformation spreading on twitter in the context of 2019 european elections. *PloS one*, *15*(1), e0227821.

Ruths, D. (2019). The misinformation machine. *Science*, *363*(6425), 348–348. Retrieved from `https://science.sciencemag.org/content/363/6425/348` doi: 10.1126/science.aaw1315

Stella, M., Ferrara, E., & De Domenico, M. (2018). Bots increase exposure to negative and inflammatory content in online social systems. *Proceedings of the National Academy of Sciences*, *115*(49), 12435–12440. Retrieved from `https://www.pnas.org/content/115/49/12435` doi: 10.1073/pnas.1803470115

[13] `https://github.com/cinhelli/Limited-Reach-Fake-News-Twitter-2019-EU-Elections`
